# A conversation with David M. Nathan, MD, Director, Diabetes Center, Massachusetts General Hospital

**DOI:** 10.1017/cts.2023.711

**Published:** 2024-01-17

**Authors:** Clinical Research Forum

**Affiliations:** Clinical Research Forum, Washington, DC, USA

## Top 10 Clinical Research Achievement Awards Q & A

This article is part of a series of interviews with recipients of Clinical Research Forum’s Top 10 Clinical Research Achievement Awards. This article is with David M. Nathan, MD, Director, Diabetes Center, Massachusetts General Hospital. Dr Nathan’s clinical research investigates novel means of controlling glycemia in the physiologic range in both Type 1 and Type 2 diabetes and the short and long-term effects of such therapy on diabetic complications. He received a 2023 Top 10 Clinical Research Achievement Award for Glycemia Reduction Approaches in Diabetes: A Comparative Effectiveness (GRADE) Study. *The interview has been edited for length and clarity.*

## When did you first become interested in clinical research?

I did a fair amount of basic science research during medical school and my early training. That was in the 1970s and benchwork was routine for anyone who was interested in academic medicine. The scope of clinical investigation, though, was rather modest at the time and mostly limited to oncology and cardiology, even at big research hospitals. Fortunately, I had a great mentor in the late 1970s and 80s, Dr Joseph Avruch, who encouraged me to focus on clinical research. He presciently recognized that these were incredibly exciting times for clinical advances in endocrinology, and especially diabetes. I went on to start the Diabetes Research Center at Massachusetts General Hospital where we helped develop the HbA1c (glycated hemoglobin) assay, the first implantable insulin pumps, and other new methodologies for treating type 1 diabetes. That work led to me being included in the Diabetes Control and Complications Trial (DCCT), which was the landmark study that demonstrated that aiming to maintain normal blood glucose concentrations reduced the development and progression of diabetes complications. The DCCT was what really got me started in clinical research. I was the youngest of the principal investigators and for me, it was like winning the World Series the first time out. It was just a spectacular study that changed the course of type 1 diabetes treatment forever.

## After the DCCT, you decided to focus more on clinical studies?

Yes. It was so exciting to see the impact of our work, and I was able to see how laboratory training and attention to designing experiments could transfer to the design and execution of clinical trials. Since then, I have been involved in and led many other studies, including the Epidemiology of Diabetes Interventions and Complications follow-up study of the DCCT, the Diabetes Prevention Program, and the GRADE study. These multi-centered studies succeed only because of the large number of talented investigators, coordinators, and, of course, our volunteer participants.

## What were the findings of the award-winning GRADE study?

The National Institute of Diabetes and Digestive and Kidney Diseases (NIDDK), National Institute of Health (NIH)-sponsored GRADE Study compared the effectiveness of four commonly used classes of glucose-lowering medications: insulin glargine U-100, the sulfonylurea glimepiride, the glucagon-like peptide-1 receptor agonist liraglutide, and sitagliptin, a dipeptidyl peptidase 4 inhibitor. A total of 5047 participants who were taking metformin for type 2 diabetes were randomly assigned to receive one of the four drugs and then followed for a mean of 5.0 years. We found that all four medications, when added to metformin, decreased glycated hemoglobin levels. However, the two injectable drugs, glargine and liraglutide, were significantly, albeit modestly, more effective in achieving and maintaining target glycated hemoglobin levels.

## Why are comparative effectiveness studies like this one so rare?

Drug companies run many trials, but those trials are typically designed to test the company’s drug versus a placebo or the “standard-of-care” to gain FDA approval. They don’t usually test their drug versus another competitor’s drug. To understand the relative benefits and risks of medications, you need to run an independent, comparative effectiveness study. It’s a credit to the NIH that they funded GRADE, a multicenter, long-duration comparative effectiveness trial. Of note, pharma did contribute substantial support in the form of donated medications. However, such studies addressing major public health questions would not be answered without NIH support.

## Where does this research go from here? What are the next steps?

We are continuing to do follow-up analyses and will publish many more papers with GRADE data. One of the most successful aspects of this trial was that we were able to recruit a diverse population (19.8% Black and 18.6% Hispanic or Latinx), so we can now look at the data across racial and ethnic groups. We are also looking at a host of baseline factors, such as demographic factors like age, sex and weight, social determinants of health, and clinical parameters including the levels of insulin sensitivity and resistance. Analyses of the relative impact of the medications on quality-of-life and health economic analyses are also ongoing. These additional analyses will help us determine more precisely who benefits more from one drug versus another.

## So ultimately, the goal is more individualized care for type 2 diabetes?

Yes. Diabetes is one of the most common chronic diseases worldwide. Type I diabetes is treated differently than type 2 diabetes, but we tend to treat all people with type 2 diabetes similarly. That’s because we still do not have great identifiers to pick out different subgroups with type 2 diabetes. Going forward, we’re interested in studying the heterogeneity of type 2 diabetes, with the hope that we can identify specific subgroups, perhaps based on genetics, metabolic profiles, or other factors or combinations of factors, that can then lead to treatment that is more personalized and potentially much more effective and efficient.

## What advice do you have for MDs who are considering clinical research?

Becoming an MD is an extraordinary accomplishment and, sometimes, it can leave you feeling like you can do anything. However, MDs who want to go into clinical research must realize that it’s a specialized discipline that is very different from patient care or other types of experimentation. Clinical research requires specific tools, and you need the right background and training, including study design, physiology, pharmacology, and data analysis. In addition, never forget that clinical research involves experiments on human beings, which adds a level of complexity that some people find frustrating – although for me it adds a dimension to the research puzzle that I find fascinating. I’d also advise MDs to make sure that they have enough protected time so that they can succeed. By that, I mean that success in clinical research rarely occurs when the research is added to full-time clinical practice. If you want to do clinical research well, you really need to dedicate yourself to it as a full-time vocation, not as an avocation. It’s not a hobby – and of course, all research is highly competitive in terms of funding. So, if you want to be able to do the research you want to do, and do it well, you need to have adequate time. You cannot give 90% to clinical and 10% to research. It just won’t work.

## What do you do outside of clinical research to “recharge your batteries?”

The first thing I would say is that I’ve been really blessed. I’ve been doing clinical research for almost 50 years, and I’ve loved every day of it. I’m a problem solver by nature and since clinical research is full of intrinsic problems, I’ve had a magical career that I’ve thoroughly enjoyed. Beyond that, I have a wonderful, supportive, loving family – my wife and two grown sons. I like to read, write, and play basketball – maybe a little slower now but still taking the “threes‘. I’ve devoted a lot of hours to my research, but when you really love something, recharging isn’t required very much.

